# Chest Pain of Suspected Cardiac Origin: Current Evidence-based Recommendations for Prehospital Care

**DOI:** 10.5811/westjem.2015.8.27971

**Published:** 2015-12-11

**Authors:** P. Brian Savino, Karl A. Sporer, Joe A. Barger, John F. Brown, Gregory H. Gilbert, Kristi L. Koenig, Eric M. Rudnick, Angelo A. Salvucci

**Affiliations:** * University of California, San Francisco, Department of Emergency Medicine, San Francisco, California; † EMS Medical Directors Association of California, California; ‡ University of California, Irvine, Center for Disaster Medical Sciences, Orange, California

## Abstract

**Introduction:**

In the United States, emergency medical services (EMS) protocols vary widely across jurisdictions. We sought to develop evidence-based recommendations for the prehospital evaluation and treatment of chest pain of suspected cardiac origin and to compare these recommendations against the current protocols used by the 33 EMS agencies in the state of California.

**Methods:**

We performed a literature review of the current evidence in the prehospital treatment of chest pain and augmented this review with guidelines from various national and international societies to create our evidence-based recommendations. We then compared the chest pain protocols of each of the 33 EMS agencies for consistency with these recommendations. The specific protocol components that we analyzed were use of supplemental oxygen, aspirin, nitrates, opiates, 12-lead electrocardiogram (ECG), ST segment elevation myocardial infarction (STEMI) regionalization systems, prehospital fibrinolysis and β-blockers.

**Results:**

The protocols varied widely in terms of medication and dosing choices, as well as listed contraindications to treatments. Every agency uses oxygen with 54% recommending titrated dosing. All agencies use aspirin (64% recommending 325mg, 24% recommending 162mg and 15% recommending either), as well as nitroglycerin and opiates (58% choosing morphine). Prehospital 12-Lead ECGs are used in 97% of agencies, and all but one agency has some form of regionalized care for their STEMI patients. No agency is currently employing prehospital fibrinolysis or β-blocker use.

**Conclusion:**

Protocols for chest pain of suspected cardiac origin vary widely across California. The evidence-based recommendations that we present for the prehospital diagnosis and treatment of this condition may be useful for EMS medical directors tasked with creating and revising these protocols.

## INTRODUCTION

The care provided by emergency medical services (EMS) varies widely in the United States. The Institute of Medicine report, “EMS at the Crossroads,” notes that an area of improvement for EMS is the need for more uniform quality care and the need to develop measures for EMS quality. 1 A major area of EMS quality that is difficult to measure is the prehospital protocols that EMS personnel follow while taking care of patients. These protocols vary widely between jurisdictions. In the state of California, EMS care is divided into 33 separate local EMS agencies (LEMSAs). These government agencies are a countywide or region-wide system of first responders and ambulance transporters that operate under one set of medical control policies.

The EMS Medical Directors Association of California (EMDAC) is a professional organization whose members include the directors of these agencies along with other interested EMS medical directors. The function of EMDAC is to provide support and guidance to the various agencies as well as to make recommendations to the California EMS Authority about policy, legislation and scope of practice issues. In an effort to improve the quality of EMS care in our state, EMDAC has endeavored to create evidence-based recommendations for EMS protocols. These recommendations are intended to assist medical directors of the various LEMSAs in developing protocols that are of high quality and evidence based. We hope to provide a summary of the evidence for the prehospital treatment of chest pain of suspected cardiac origin and to measure the consistency of current California protocols.

## METHODS

A subcommittee of EMDAC endeavored to create a narrative review of the existing evidence for prehospital treatment of chest pain. The subcommittee chose by consensus the elements that should be included in any protocol for chest pain of suspected cardiac origin.

Clinical questions regarding these interventions were developed in the population, intervention, control group and outcome format. Our population was those patients in the prehospital setting with chest pain of suspected cardiac origin. The intervention varied by clinical question. The control group consisted of patients who were not receiving the specific intervention, and outcomes were defined by resolution of electrocardiographic (12-lead ECG) findings, chest pain resolution, infarct size and mortality. The outcomes varied considerably depending on the individual study design. These commonly included cardiac events, rate of myocardial infarction (MI), arrhythmias, shock, death, length of stay, infarct size, need for percutaneous intervention (PCI) and/or ejection fraction.

We relied heavily on recommendations made by various organizations that have performed systematic reviews and meta-analyses regarding these treatment interventions including the American Heart Association (AHA), the Cochrane Group and the International Liaison Committee on Resuscitation (ILCOR). We supplemented the recommendations from these organizations with additional literature searches through PubMed for each specific question.

The process used for assigning levels of evidence (LOE) and grading our recommendations was taken from the American College of Emergency Physicians (ACEP) process of creating their clinical policies with slight modification to better fit our objectives. A committee of EMDAC reviewed studies and assigned LOE based on the study design, including features such as data collection methods, randomization, blinding, outcome measures and generalizability. 2 A brief summary of the reviewed studies is available in an electronic appendix. LOE I consisted of randomized, controlled trials, prospective cohort studies, meta-analysis of randomized trials or prospective studies, or clinical guidelines/comprehensive review. LOE II consisted of nonrandomized trials and retrospective studies. LOE III consisted of case series, case reports, and expert consensus. After assigning LOE to the studies, these were translated to clinical grades of our recommendations using the following standards:

### Level A recommendations

Prehospital recommendations with a strong degree of certainty based on one or more LOE I studies or multiple LOE II studies.

### Level B recommendations

Prehospital recommendations with a moderate degree of certainty based on one or more LOE II studies or multiple LOE III studies.

### Level C recommendations

Prehospital recommendations that are based on only poor quality or minimal LOE III studies or based on consensus.

### No recommendation

No recommendation will be given in those cases where only preliminary data or no published evidence exists and we have no expert consensus. We may also withhold recommendation when studies, no matter their LOE, currently show conflicting data.

After answering the clinical question and providing recommendations for diagnostic and treatment interventions, each current chest pain protocol for the 33 agencies were reviewed for consistency with the recommendations. The clinical protocols were reviewed during the month of July 2015. Institutional review board approval was deemed to not be necessary for this review of publicly available research and clinical protocols.

### Supplemental Oxygen

#### Clinical Question

Does the prehospital administration of oxygen to patients with chest pain and normal oxygen saturations improve outcomes in cases of suspected acute coronary syndrome (ACS)?

#### Summary of Current Evidence

There have been few randomized controlled studies that have attempted to answer this question. A study in 1976 by Rawles et al. randomized patients to oxygen or air and found more deaths in the oxygen group, although not clinically significant. 3 A more recent trial addressing this question was in 2012 by Ranchord et al. showing no difference in mortality between a titrated oxygen group and a high-flow oxygen group, but with a very small sample size. 4 The ILCOR ACS guidelines in 2010 do not find sufficient evidence to support the use of oxygen in suspected ACS, but do not find evidence of harm. 5 A meta-analysis by Cochrane review (updated in 2013) showed no evidence of benefit and, in fact, showed possible harm with routine oxygen administration in suspected ACS, but noted that the analysis lacked the power to substantiate or refute the use of oxygen in these cases. 6 A recent multicenter, prospective, randomized, controlled trial compared prehospital oxygen (8L/min) with no supplemental oxygen in patients with ST segment myocardial infarction (STEMI) and oxygen saturation of 94% or greater. 7 The authors demonstrated that supplemental oxygen in this group increased early myocardial injury and was associated with larger myocardial infarct size and a higher rate of re-infarction. The DETO2X-AMI trial is ongoing and will examine this question among patients with suspected ACS and should be adequately powered to address this question. 8

#### Current Prehospital Treatment Recommendation

There is no evidence that the routine use of oxygen to patients with normal oxygen saturations provides any benefit, but may cause harm.

##### Level A Recommendation

We recommend against routine oxygen supplementation in normoxic patients (oxygen saturation of 94% or greater) with suspected ACS. 9 We recommend using supplemental oxygen for those patients with suspected ACS and signs of heart failure or shock. 9

##### Level B Recommendation

None given.

##### Level C Recommendation

Oxygen saturations should be titrated between 94%-98% (consensus).

### Aspirin

#### Clinical Question

Does the prehospital administration of aspirin to patients with suspected ACS improve outcomes with an acceptable rate of adverse events?

#### Summary of Current Evidence

There is high-quality evidence demonstrating benefit of aspirin administration (162.5mg) in improving mortality among patients with an acute myocardial infarction (MI). 5 - 11 This reduction in long-term mortality is greatest when the aspirin is administered early. 12,13

#### Current Prehospital Treatment Recommendation

##### Level A Recommendation

We recommend the administration of aspirin to adults with chest pain due to suspected ACS. In making this recommendation, we place a higher value on the benefits of aspirin (decreased mortality and decreased complications of MI), which outweigh the risks of adverse effects (gastrointestinal bleeding). Aspirin is contraindicated in the setting of known aspirin allergy.

##### Level B Recommendation

None given.

##### Level C Recommendation

None given.

### Nitrates

#### Clinical Question

Does the administration of nitroglycerin in the prehospital setting to patients with suspected ACS improve outcomes when compared to not using nitrates?

#### Summary of Current Evidence

There have been no trials to specifically evaluate the usefulness of nitrates in the field or in the emergency department (ED) among patients with chest pain of suspected ACS. 5 A reduction in infarct size (using creatinine kinase as a surrogate measure) was noted in those treated within three hours of symptoms in three studies of intensive care unit patients. 14–16 There have been two trials showing that combined treatment with nitroglycerin and fibrinolytics may have a detrimental effect on reperfusion. 17,18 There is currently not enough evidence to suggest clinical benefit or harm of nitroglycerin use in the prehospital setting.

#### Current Prehospital Treatment Recommendation

##### Level A Recommendation

None given.

##### Level B Recommendation

None given.

##### Level C Recommendation

If nitroglycerin is used, then the following contraindications should be part of the protocol (expert consensus):

- Contraindications with hypotension, defined as a systolic blood pressure less than 90mmHg, by expert consensus.
- Contraindications with suspected right side/inferior infarct.
- Contraindication with the recent use of phosphodiesterase-5 inhibitors for erectile dysfunction or pulmonary hypertension.

##### No recommendation

Although it is reasonable to consider the early administration of nitroglycerin in selected patients without contraindications, insufficient evidence exists to support or refute the routine administration of nitroglycerin in the ED or prehospital setting in patients with suspected ACS. 5

### Opiates

#### Clinical Question

Does the administration of opiates to patients with suspected ACS improve outcomes when compared to not using opiates?

#### Summary of Current Evidence

Clear benefit to opiate administration is unclear. There is a single study that suggested mortality and rates of infarction are more prevalent in patients who receive morphine with Non STEMI. 5,19 No studies have been done with fentanyl among patients with chest pain of suspected cardiac etiology.

#### Current Prehospital Treatment Recommendation

##### Level A Recommendation

None given.

##### Level B Recommendation

None given.

##### Level C Recommendation

If opiates are used, they should be administered intravenously and titrated to pain relief (consensus). Contraindications with hypotension, defined as a systolic blood pressure less than 90mmHg.

##### No Recommendation

Although it is reasonable to consider the early administration of opiates in selected patients without contraindications, insufficient evidence exists to support or refute its routine administration in the ED or prehospital setting in patients with chest pain of suspected ACS. There is only one poor quality study that demonstrated harm.

### 12-lead ECG

#### Clinical Question

Does the prehospital use of a 12-lead ECG increase the diagnostic sensitivity and specificity of STEMI among patients with chest pain of suspected ACS when compared to not using a prehospital 12-lead ECG? 5

#### Summary of Current Evidence

Several studies have demonstrated that prehospital 12-lead ECGs can improve the recognition of STEMI with reasonable sensitivity and specificity. 20–26 Repeat prehospital or ED 12-lead ECGs may be helpful. 21,27 The timely notification of the STEMI center is helpful in reducing door-to-intervention times. 28 The research on computer-interpreted electrocardiography has been mixed but seems to be generally accurate and had a greater influence on non-expert performance. 5,29 There has been limited research in the effectiveness of transmission of the 12-lead ECG. 30

#### Current Prehospital Treatment Recommendation

##### Level A Recommendation

In patients with suspected ACS, a 12-lead ECG should be acquired and interpreted by prehospital or emergency providers as soon as possible after first patient contact. The interpretation should be used in conjunction with the clinical signs and presentation for diagnosis and triage, including destination decisions.

##### Level B Recommendation

Repeated prehospital 12-lead ECGs may improve diagnostic accuracy of STEMI. Transmitted 12-lead ECGs may be useful in decreasing door-to-intervention times. The timely notification of the STEMI-receiving center is helpful in decreasing door-to-intervention times. Prehospital activation of the catheterization lab may also help to decrease door-to-intervention times. Computer interpretation of 12-lead ECGs may help to increase the specificity of the diagnosis, especially with less experienced paramedics.

##### Level C Recommendation

None given.

### Regionalization of STEMI Care

#### Clinical Question

Does the regionalization of STEMI care lead to decreased door-to-intervention times and improved patient outcomes in prehospital patients with STEMI when compared to a non-regionalized program?

#### Summary of Current Evidence

It has been shown in multiple studies that primary PCI is the ideal method of reperfusion in patients presenting with STEMI. 31 Timely PCI leads to decreased morbidity and mortality in this patient population. 32,33 Current AHA recommendations call for a first medical contact to intervention time of less than 90 minutes and additionally note that the EMS system can play a large role in decreasing not only D2B time, but “total ischemic time,” as well. 11,34,35 The AHA also recognizes that PCI is not always available and in these cases thrombolytics may be required. 11 Regionalization of STEMI care does lead to decreased door-to-intervention times. 36,37 The evidence for improvements in mortality and other clinical outcomes among STEMI patients are less well studied.

Rapid inter-facility transfers of patients with STEMI presenting to a non-PCI hospital can reduce time to treatment. STEMI systems should include an organized inter-facility transfer process that includes inter-hospital agreements and ambulance dispatch protocols designed to minimize transfer time.

#### Current Prehospital Treatment Recommendation

##### Level A Recommendation

There is a large body of evidence suggesting that primary PCI is superior to thrombolytics. There is also evidence suggesting benefit of STEMI regionalization programs in decreasing door-to-intervention times. We recommend EMS systems employ a regionalization program for STEMI patients that provide direct transport to PCI capable centers. In cases where timely transport to a PCI center is not possible (>90min), transport to a facility that can provide thrombolytic therapy is reasonable.

##### Level B Recommendation

None given.

##### Level C Recommendation

None given.

### Fibrinolytics

#### Clinical Question

In patients with STEMI and a prolonged time to primary PCI, does the use of prehospital fibrinolytics improve outcomes?

#### Summary of Current Evidence

There is a significant body of high-quality evidence describing the benefit of fibrinolytic therapy given to patients with STEMI when it is anticipated that primary PCI cannot be performed within 120 minutes of first medical contact. 10,11,38,39 If prehospital fibrinolysis is chosen as the reperfusion strategy, there should be well-established protocols with a competency training program, performance improvement, and robust medical oversight. This is not currently in the scope of practice for paramedics in the state of California.

#### Current Prehospital Treatment Recommendation

##### Level A Recommendation

None given.

##### Level B Recommendation

None given.

##### Level C Recommendation

For those patients with a STEMI, onset of ischemic symptoms within the previous 12 hours and primary PCI cannot be accomplished within 120 minutes of first medical contact, prehospital fibrinolytics can be considered. There should be considerable oversight for any prehospital thrombolytic program. Because it is not in the current scope of practice for paramedics in California, this could only be done as a pilot study with approval from the EMS Authority.

### β-Blockers

#### Clinical Question

Does the prehospital administration of a β-blocker to patients with an acute STEMI improve outcomes?

#### Summary of Current Evidence

Current recommendations by the AHA are that a β-blocker be administered in the first 24 hours after an acute MI. 40 There is evidence to show that early β-blocker administration may improve infarct size and left ventricular ejection fraction. 41 There has been one study comparing prehospital metoprolol administration to patients with STEMI to those receiving metoprolol 12 to 24 hours later. The prehospital patients, in this study, showed smaller infarct sizes and improved ejection fractions. 42

#### Current Prehospital Treatment Recommendation

##### Level A Recommendation

None given.

##### Level B Recommendation

None given.

##### Level C Recommendation

None given.

##### No Recommendation

While the prehospital administration of β-blockers does seem to show promise, there are insufficient studies at this time to make a prehospital treatment recommendation regarding their use in the field.

## RESULTS

All 33 agencies protocols were identified and reviewed for consistency with the recommendations made by EMDAC for chest pain of suspected cardiac origin. Every agency has a protocol relating to the treatment of chest pain, though these protocols vary significantly in content and organization. Examples of suggested language for protocol development that the committee felt was most consistent with the recommendations were taken from the agency protocols.

### Supplemental Oxygen Administration

Every agency has a component of oxygen administration in their protocol for chest pain. Routine use of oxygen, regardless of the patient’s oxygen saturation is recommended in 39% of agencies, with 54% percent of agencies advising a titrated dose to their providers (Table 1). Only four of the agencies had language specifically advising against the use of supplemental oxygen if the patient had normal oxygen saturation. All agencies advise the use of supplemental oxygen in cases of shock or respiratory distress, even if the patient has normal oxygen saturations.

### Aspirin Administration

The use of aspirin for chest pain of suspected cardiac etiology is universal among our agencies (Table 2). There is no consensus on the dose of aspirin to be used. Sixty-four percent of agencies recommend a 324mg dose, 24% recommend a 162mg dose and 15% recommend either 162mg or 325mg dosing.

There is also great variability in what are considered contraindications to aspirin use. Aspirin allergy is specifically noted as a contraindication in 58% of agencies and recent gastrointestinal bleeding is noted in 30% of agencies. Two agencies noted peptic ulcer disease as a contraindication and one noted asthma, chest pain radiating to the mid back and chronic anticoagulant use as contraindications. Some protocols clarify that aspirin is indicated in the setting of the use of other anticoagulants (e.g. warfarin) or of gastrointestinal disease without a recent bleeding episode.

### Nitroglycerin Administration

All agencies include sublingual nitroglycerin, either tablets or spray, in the treatment of chest pain (Table 3). One agency does not allow the use of nitroglycerin for patients with STEMI. Topical nitroglycerin paste is present in 36% of the protocols. Hypotension is noted as a contraindication to nitroglycerin use in 100% of the protocols; however, the definition of hypotension varies. A systolic blood pressure as less than 90mmHg is the definition of hypotension in 36% of agencies while less than 100mm Hg is the definition in 52% of agencies. One agency (2%) uses less than 110mmHg as the cut off and three agencies (9%) do not define hypotension. Right-sided or inferior MI is a contraindication in 21% of agencies and phosphodiesterase 5 (PDE5) inhibitor use is noted as a contraindication in 91% of protocols.

### Opiate Administration

Opiate use for chest pain is recommended in all protocols. Morphine sulfate is the opiate used in 58% of agencies, while fentanyl is the opiate of choice in 15% of agencies (Table 4). Both medications are available in 27% of the agencies’ protocols. Hypotension is noted as a contraindication for 88% of the protocols. Of those that mention hypotension as a contraindication for opiate administration, 41% define it as a systolic blood pressure of less than 90mmHg and 45% define it as less than 100mmHg. One agency (3%) defines it as less than 110mmHg, and three agencies (10%) do not define hypotension.

### Prehospital 12-lead ECGs

Prehospital 12-lead ECGs are used in all but one of the 33 agency (97%) (Table 5). Transmission of the ECG to a receiving facility is advised in 61% of the protocols. Computer interpretation of the ECG is mentioned in 82% of the protocols and some form of medic interpretation (whether by noting morphology or contacting the base hospital) is mentioned in 42% of protocols. Timely receiving center notification of STEMI is mandated in 94% of protocols. Serial 12-lead ECGs during transport of a patient with chest pain is practiced by 55% of agencies.

### STEMI Regionalization

All but one (97%) of the agencies has some form of regionalization of STEMI care. These systems are not uniform in design and the capabilities of the systems vary widely based on geography and access to PCI capable receiving centers.

### Prehospital Fibrinolytics

There are currently no agencies that employ fibrinolytics in the field. One agency received approval in the past for a trial study of prehospital fibrinolytics for STEMI patients but enrolled no patients during the trial study period.

## DISCUSSION

The chest pain protocols reviewed varied greatly in content and structure between LEMSAs in California. These government agencies consist of either a county or region that develops a system of care that include first responders, ambulance transporters, and specialty receiving facilities. These systems reflect the needs and demographics of that county or region and operate under one set of medical control policies.

A similar variation among protocols was seen in a recent study on state wide EMS protocols. 43 The National Association of EMS Officials recently published model EMS guidelines that could be used to decrease this variability. 44

Many agencies use some sort of titration language for oxygen use but only four restricted use with normal saturations. The current evidence seems to point towards more restrictive language for our protocols. Aspirin use, while universal, varied considerably in dosing and many agencies did not have contraindications clearly noted in their protocols. Local training could address these issues, even if not explicitly stated in the protocols themselves. Recent studies have demonstrated low rates of prehospital aspirin administration despite being almost universal in our protocols. 45

Nitroglycerin is used by every agency with a wide variability noted in delivery route. Few agencies noted a contraindication with right-sided infarcts, with one agency not allowing nitrates in any patient with STEMI to avoid this potential contraindication. Most agencies did caution use with PDE5 Inhibitors. Holding nitroglycerin for hypotension was universal, but the systolic blood pressure cut-off varied. Our recommended cut-off is a systolic blood pressure of 90mmHg.

Opiates were also found to be used in all protocols with many agencies preferring fentanyl over morphine. It should be noted that studies with fentanyl for ischemic chest pain are not available to specifically recommend this agent for suspected ACS.

Twelve-lead ECGs were used in all but one agency. The technique used to interpret STEMI varied between agencies with some using computer interpretation, medic interpretation or both. Most agencies notified receiving centers of their potential STEMI and many have transmission capabilities.

Prehospital fibrinolytics, while not employed at any agency, may still have a place for some rural systems without timely access to regionalized STEMI centers. Currently, paramedics in California do not have this in their scope of practice. If such a system were to be put in place it would require a trial study and substantial medical oversight.

While many states have mandatory protocols in place for their providers, California has allowed the individual agencies to develop and implement their own protocols. While this has allowed for flexibility among regions with different populations and financial and geographic restrictions, it may also contribute to health outcome disparities among the agencies. A study by Kupas et al. coined the term “model” protocols, which were found to be present in 17 states. 43 In contrast with mandatory protocols, which require local agencies to adopt the state protocols, model protocols are in place as an option for local use, but not required. This respects the local agencies’ autonomy, but also provides a standard that can be adopted should a local agency need to create or change its protocols.

## CONCLUSION

Protocols for chest pain of suspected cardiac origin vary widely across the state of California. The evidence-based recommendations that we present for the prehospital diagnosis and treatment of this condition may be useful for EMS medical directors tasked with creating and revising these protocols.

## Supplementary Information



## Figures and Tables

**Table 1:** Oxygen use in patients with chest pain.

LEMSA	Routine use regardless of SpO_2_%	Oxygen titration	Titration range	Advise against use with normal SpO_2_%	Advise use in shock or dyspnea
Alameda County	No	Yes	94–99%	No	Yes
Central California	Yes	No	N/A	No	Yes
San Francisco	No	Yes	94–95%	No	Yes
Coastal Valleys	No	Yes	94–98%	No	Yes
Contra Costa County	No	Yes	>94%	No	Yes
El Dorado County	Yes	No	N/A	No	Yes
Imperial County	No	Yes	94%	No	Yes
Inland Counties	No	No	N/A	No	Yes
Kern County	Yes	No	N/A	No	Yes
Los Angeles County	No	Yes	94–98%	No	Yes
Marin County	No	Yes	94–99%	No	Yes
Merced County	Yes	No	N/A	No	Yes
Monterey County	No	No	N/A	No	Yes
Mountain Valley	Yes	No	N/A	No	Yes
Napa County	Yes	Yes	>94%	No	Yes
Northern California	Yes	No	N/A	No	Yes
North Coast	No	Yes	>94%	Yes	Yes
Orange County	No	Yes	>94%	No	Yes
Riverside County	No	Yes	>94%	No	Yes
Sacramento County	No	Yes	95%	Yes	Yes
San Benito County	No	Yes	95%	Yes	Yes
San Diego County	No	No	N/A	No	Yes
San Joaquin County	Yes	No	N/A	No	Yes
San Luis Obispo County	Yes	No	N/A	No	Yes
San Mateo County	Yes	No	N/A	No	Yes
Santa Barbara County	No	Yes	94%	No	Yes
Santa Clara County	Yes	No	N/A	No	Yes
Santa Cruz County	No	Yes	94–95%	Yes	Yes
Sierra-Sacramento	Yes	Yes	>94%	No	Yes
Solano County	Yes	No	N/A	No	Yes
Tuolumne County	No	No	N/A	No	Yes
Ventura County	No	Yes	94%	No	Yes
Yolo County	No	Yes	94–99	No	Yes

*LEMSA,* local emergency medical services angencies

**Table 2:** Aspirin use in patients with chest pain.

LEMSA	Used	Dose (mg)	Hold if allergy	Hold if recent GI bleed
Alameda County	Yes	162–324	No	No
Central California	Yes	162	Yes	Yes
San Francisco	Yes	324	Yes	Yes
Coastal Valleys	Yes	324	Yes	Yes
Contra Costa County	Yes	325	Yes	Yes
El Dorado County	Yes	162	Yes	No
Imperial County	Yes	162	No	No
Inland Counties	Yes	324/325	No	No
Kern County	Yes	325	Yes	Yes
Los Angeles County	Yes	162–325	Yes	Yes
Marin County	Yes	162–325	No	No
Merced County	Yes	324	Yes	Yes
Monterey County	Yes	162	No	No
Mountain Valley	Yes	324	Yes	No
Napa County	Yes	162	Yes	Yes
Northern California	Yes	324/325	Yes	Yes
North Coast	Yes	324	Yes	No
Orange County	Yes	324	Yes	No
Riverside County	Yes	324	No	No
Sacramento County	Yes	325	Yes	No
San Benito County	Yes	162	Yes	No
San Diego County	Yes	324	No	No
San Joaquin County	Yes	325	Yes	Yes
San Luis Obispo County	Yes	162	Yes	Yes
San Mateo County	Yes	324	Yes	No
Santa Barbara County	Yes	324	No	No
Santa Clara County	Yes	324	No	No
Santa Cruz County	Yes	162	Yes	No
Sierra-Sacramento	Yes	325	No	No
Solano County	Yes	325	No	No
Tuolumne County	Yes	325	No	No
Ventura County	Yes	324	No	No
Yolo County	Yes	162–325	Yes	No

*LEMSA,* local emergency medical services agency; *GI*, gastrointestinal

**Table 3:** Nitroglycerin use in patients with chest pain.

LEMSA	Used	Route	Hold if hypotension	Hold if right or inferior MI	Hold if PDE5 inhibitor
Alameda County	Yes	SL	Yes (90/30)	Yes	Yes
Central California	Yes	SL, Paste	Yes (<100sys)	No	No
San Francisco	Yes	SL	Yes (<90sys)	Yes	Yes
Coastal Valleys	Yes	SL, Paste	Yes (<100sys)	No	Yes
Contra Costa County	Yes	SL	Yes (<90sys)	All STEMI	Yes
El Dorado County	Yes	SL, Paste	Yes (<100sys)	No	Yes
Imperial County	Yes	SL	Yes (<100sys)	No	Yes
Inland Counties	Yes	SL	Yes	Yes	Yes
Kern County	Yes	SL	Yes (<90sys)	No	Yes
Los Angeles County	Yes	SL	Yes (<100sys)	No	Yes
Marin County	Yes	SL	Yes (<100sys)	Yes	Yes
Merced County	Yes	SL, Paste	Yes (<100sys)	No	Yes
Monterey County	Yes	SL, Paste	Yes (<110sys)	No	Yes
Mountain Valley	Yes	SL	Yes (<100sys)	No	Yes
Napa County	Yes	SL, Paste	Yes (<100sys)	No	Yes
Northern California	Yes	SL	Yes (<90sys)	No	Yes
North Coast	Yes	SL	Yes (<90sys)	Yes	No
Orange County	Yes	SL	Yes (<90sys)	No	Yes
Riverside County	Yes	SL, Paste	Yes (<90sys)	No	Yes
Sacramento County	Yes	SL	Yes (<90sys)	No	Yes
San Benito County	Yes	SL, Paste	Yes	No	Yes
San Diego County	Yes	SL, Paste	Yes (<100sys)	No	Yes
San Joaquin County	Yes	SL	Yes (<90sys)	No	Yes
San Luis Obispo County	Yes	SL, Paste	Yes (<100sys)	Yes	Yes
San Mateo County	Yes	SL	Yes (<90sys)	No	Yes
Santa Barbara County	Yes	SL	Yes (<100sys)	No	Yes
Santa Clara County	Yes	SL	Yes (<100sys)	No	Yes
Santa Cruz County	Yes	SL, Paste	Yes	No	Yes
Sierra-Sacramento	Yes	SL	Yes (<100sys)	No	Yes
Solano County	Yes	SL	Yes (<100sys)	No	Yes
Tuolumne County	Yes	SL	Yes (<90sys)	No	Yes
Ventura County	Yes	SL	Yes (<100sys)	No	Yes
Yolo County	Yes	SL, Paste	Yes (<100sys)	Yes	Yes

*LEMSA,* local emergency medical services angencies; *MI*, myocardial infarction, *STEMI,* ST segment elevation myocardial infarction; *SL*, sublingual; *sys*, systolic

**Table 4:** Opiate use in patients with chest pain.

LEMSA	Used	Opiate	Route	Hold if hypotension
Alameda County	Yes	Fentanyl	IV/IO/IM/IN	Yes (<90sys)
Central California	Yes	Fentanyl	IV/IN	Yes (<100sys)
San Francisco	Yes	Morphine	IV/IO/IM	Yes (<90sys)
Coastal Valleys	Yes	Fentanyl	IV	Yes (<100sys)
Contra Costa County	Yes	Fentanyl	IV	Yes (<90sys)
El Dorado County	Yes	Fentanyl/MS	IV/IO/IM/IN	Yes (<90sys)
Imperial County	Yes	Morphine	IV	Not mentioned
Inland Counties	Yes	Fentanyl/MS	IV/IO/IM/IN	Not mentioned
Kern County	Yes	Morphine	IV	Yes (<90sys)
Los Angeles County	Yes	Fentanyl/MS	IV/IO	Yes
Marin County	Yes	Morphine	IV	Not mentioned
Merced County	Yes	Morphine	IV	Yes (<100sys)
Monterey County	Yes	Morphine	IV	Yes (<110sys)
Mountain Valley	Yes	Morphine	IV/IO/IM	Yes (<100sys)
Napa County	Yes	Fentanyl	IV/IN	Yes (<100sys)
Northern California	Yes	Morphine	IV	Not mentioned
North Coast	Yes	Morphine	IV/IM	Yes (<90sys)
Orange County	Yes	Fentanyl/MS	IV	Yes (<90sys)
Riverside County	Yes	Morphine	IV/IO/IM	Yes (<90sys)
Sacramento County	Yes	Fentanyl/MS	IV/IO	Yes (<90sys)
San Benito County	Yes	Morphine	IV/IO	Yes
San Diego County	Yes	Morphine	IV	Yes (<100sys)
San Joaquin County	Yes	Morphine	IV	Yes (<90sys)
San Luis Obispo County	Yes	Morphine	IV	Yes (<100sys)
San Mateo County	Yes	Morphine	IV/IM	Yes (<90sys)
Santa Barbara County	Yes	Morphine	IV/IM	Yes (<100sys)
Santa Clara County	Yes	Morphine	IV	Yes (<100sys)
Santa Cruz County	Yes	Morphine	IV/IO	Yes
Sierra-Sacramento	Yes	Fentanyl/MS	IV/IO	Yes (<100sys)
Solano County	Yes	Fentanyl/MS	IV	Yes (<100sys)
Tuolumne County	Yes	Fentanyl/MS	IV	Yes (<90sys)
Ventura County	Yes	Morphine	IV/IM	Yes (<100sys)
Yolo County	Yes	Fentanyl/MS	IV/IO/IM	Yes (<100sys)

*LEMSA,* local emergency medical services angencies; *IV*, intravenous; *IM*, intramuscular; *IO*, intraosseous; *sys*, systolic; *MS*, morphine sulfate

**Table 5:** 12-lead ECG in patients with chest pain.

LEMSA	Used	Transmit	Computer interprets	Medic interprets	Notify STEMI center	Serial ECGs
Alameda County	Yes	Yes	Yes	Yes	Yes	Yes
Central California	Yes	No	Yes	No	Yes	No
San Francisco	Yes	Yes	No	Yes	Yes	Yes
Coastal Valleys	Yes	Yes	Yes	Yes	Yes	Yes
Contra Costa County	Yes	Yes	Yes	No	Yes	Yes
El Dorado County	Yes	Yes	Yes	No	Yes	No
Imperial County	Yes	No	Unclear	Unclear	Yes	No
Inland Counties	Yes	No	No	Yes	Yes	Yes
Kern County	Yes	Yes	Yes	No	Yes	No
Los Angeles County	Yes	Yes	Yes	No	Yes	No
Marin County	Yes	Yes	Yes	Yes	Yes	No
Merced County	Yes	No	Yes	No	Yes	No
Monterey County	Yes	Yes	Yes	Yes	Yes	Yes
Mountain Valley	Yes	No	Yes	No	Yes	Yes
Napa County	Yes	Yes	Yes	Yes	Yes	Yes
Northern California	Yes	Yes	Unclear	Yes	Yes	No
North Coast	Yes	No	Yes	Yes	Yes	Yes
Orange County	Yes	No	Yes	No	Yes	No
Riverside County	Yes	Yes	Yes	Yes	Yes	No
Sacramento County	Yes	No	Yes	No	Yes	No
San Benito County	No	No	No	No	No	No
San Diego County	Yes	No	Yes	No	No	Yes
San Joaquin County	Yes	Yes	Yes	No	Yes	No
San Luis Obispo County	Yes	No	Yes	Yes	Yes	Yes
San Mateo County	Yes	Yes	Yes	Yes	Yes	Yes
Santa Barbara County	Yes	No	Yes	No	Yes	Yes
Santa Clara County	Yes	Yes	Yes	Yes	Yes	No
Santa Cruz County	Yes	Yes	No	Yes	Yes	No
Sierra-Sacramento	Yes	Yes	Yes	No	Yes	No
Solano County	Yes	Yes	Yes	No	Yes	Yes
Tuolumne County	Yes	Yes	Yes	No	Yes	No
Ventura County	Yes	No	Yes	No	Yes	Yes
Yolo County	Yes	Yes	Yes	Yes	Yes	No

*LEMSA,* local emergency medical services angencies; *ECG*, electrocardiogram; *STEMI,* ST segment elevation myocardial infarction
