# Supplementary material for: Chest Pain of Suspected Cardiac Origin: Current Evidence-based Recommendations for Prehospital Care
Source: West J Emerg Med. 2015 Dec 11;16(7):983–95. doi: 10.5811/westjem.2015.8.27971 (PMC4703143; doi:10.5811/westjem.2015.8.27971)
Supplement: Supplementary file 1 [file wjem-16-983-s001.docx]

**Evidentiary Table: Oxygen**

| **Study** | **LOE** | **Study Design** | **Methods and Outcomes** | **Results** | **Limitations** |
| --- | --- | --- | --- | --- | --- |
| Rawles 1976[^3^](#_ENREF_3) | I | Double-blinded, randomized controlled trial | 200 people presenting 24 hours after AMI were randomized to oxygen vs. compressed air. Outcomes included death, length of stay, pain medication need, arrhythmias, systolic ejection times. | More deaths in O^2^ group, though non-significant p-value. No significant difference in systolic ejection times or arrhythmias (except sinus tachycardia in oxygen group.) | Early study, unable to measure important data like infarct size. Adequacy of blinding is unclear. |
| Ranchord 2012[^4^](#_ENREF_4) | I | Randomized controlled trial | 148 people presenting with ACS symptoms with STEMI findings on EKG or new left bundle. Randomized to high concentration vs titrated oxygen (93-96%). Outcomes included death at 30 days, complications, infarct size and mass, re-infarction and revascularization at 30 days. | No statistical significance for death, troponin, LV function, infarct mass or percent infarct mass between titrated and high concentration oxygen. | Oxygen delivered to some participants prior to being randomized. Not all participants received MRI imaging. Study was not blinded. |
| Stub 2015[^7^](#_ENREF_7) | I | Randomized controlled trial | 638 patients randomized to air (given O2 if saturations dropped below 94%) vs oxygen (8L via facemask regardless of saturation). Randomization occurred in the prehospital setting. Outcomes included infarct size (assessed by troponin and CK), recurrent MI, cardiac arrhythmia and MI size at 6 months. | O^2^ group: Increase in mean peak CK, recurrent MI, cardiac arrhythmia and increase in infarct size when compared to air, all statistically significant. | Providers not blinded to therapy. |
| O’Connor 2010 for ILCOR[^5^](#_ENREF_5) | I | Comprehensive Review | Structured review with goal to create guidelines for ACS care. 25 topics for review were identified, including oxygen therapy. | Four studies identified looking at O2 therapy in acute MI. No evidenced found for or against use. | Not original research. Not able to give recommendation on oxygen use. |
| Cabello 2013 for Cochrane[^6^](#_ENREF_6) | I | Meta-analysis and Comprehensive Review | Structured database review searching for randomized controlled trials with suspected or proven acute MI looking at benefit of inhaled oxygen vs air. Outcomes included pain and death. | Four trials with 430 participants identified. 17 deaths. Relative risk of death identified as 2.05, not shown to be confirmatory for harm, though no clear benefit. | Small number of studies. |

**Evidentiary Table: Aspirin**

| **Study** | **LOE** | **Study Design** | **Methods and Outcomes** | **Results** | **Limitations** |
| --- | --- | --- | --- | --- | --- |
| O’Connor  2010 for  ILCOR[^5^](#_ENREF_5) | I | Comprehensive Review | Structured database review with the goal of creating guidelines for ACS care. 25 topics were selected for review including the use of aspirin in ACS and the role for prehospital administration. | Two studies identified showing clear benefit to ASA therapy. Two studies showing benefit of EMS use outweighs harm. One study showing no benefit to EMS use. Recommends dispatch or EMS administration if no allergy or bleeding disorder. | Very few prehospital studies identified to make strong recommendation for EMS use. |
| ISIS-2 Coll. Group  1988[^10^](#_ENREF_10) | I | Double-blinded randomized controlled trial | 17,187 patients at 417 hospitals presenting with acute MI were randomized to streptokinase, aspirin or both (or placebo of all three options). Outcomes: Vascular death at 5 weeks, reinfarction, cardiac rupture, cardiac arrest, bleeding, stroke and non-vascular death. Side-effects also monitored. | Aspirin showed significant survival over placebo (9.4% v. 11.8%) with combination aspirin and streptokinase showing greatest survival benefit (8% v. 13.2%). |  |
| O’Gara 2013 for the AHA[^40^](#_ENREF_40) | I | Comprehensive Review/Clinical Guideline | Structured review on the treatment of ST-elevation MI, including the role of aspirin use in suspected cases. | Three studies identified leading to a Class I recommendation by the AHA for aspirin administration prior to PCI. | These are clinical guidelines based on original research. Does not specify the role of EMS in administration of aspirin. |
| Freimark 2002[^12^](#_ENREF_12) | I | Subset Analysis of double-blinded randomized controlled trial (ARGAMI-2 trial) | Original study was looking at heparin vs. argatroban in MI patients receiving lytics. The timing of aspirin administration was recorded during the study and subset analysis performed on 1200 patients. Early administration defined as before thrombolytic therapy. Late administration was defined as after thrombolysis. Outcomes: Mortality (7, 30 and 365 days), recurrent MI, recurrent ischemia,  v-fib/tach, shock. | Median time to ASA for early vs late: 1.6 vs 3.5 hours. Mortality was significantly lower in early group at 7 days (2.5% vs 5.6%), 30 days (3.3% vs 7.3%) and 1 year (5% vs 10.6%), all with significant p-values. | Subset analysis. No clearly defined time for early vs. late administration. |
| Barbash  2002[^13^](#_ENREF_13) | III | Case Series | Using data from a national Israeli survey (ACSIS 200) 922 consecutive patients with AMI were identified. They were divided into two groups: early (prior to admission) vs. late (after admission) ASA administration. Outcomes: Mortality at 7 and 30 days, in-hospital complications. | Early administration associated with lower mortality at 7 (2.4 vs 7.3% p=0.002) and 30 (4.9 vs. 11.1% p=0.001) days with lower in-hospital complications. | No time-frame defined for early vs late. No randomization or blinding. |

**Evidentiary Table: Nitroglycerin**

| **Study** | **LOE** | **Study Design** | **Methods and Outcomes** | **Results** | **Limitations** |
| --- | --- | --- | --- | --- | --- |
| Bussman 1981[^14^](#_ENREF_14) | II | Non-randomized trial | Patients chosen with acute MI and PCWP over 15 mm Hg. 29 patients in control group. 31 patients received a nitroglycerineinfusion over 48 hours. All received a Swan-Ganz Outcomes: Infarct size (defined by CK/CK-MB index), Cardiac output, Left ventricular filling pressures | Rate of CK release reduced from 79 to 33 U/hr in nitrate group with calculated infarct size 48 gEq in nitrate group and 69 gEq in control. Nitrate group also had greater cardiac output and lower left ventricular filling pressures. | Non-blinded study. Randomization process is unclear, if any. Small sample size. Early study unable to calculate infarct size directly, using CK as a surrogate. |
| Charvat 1990[^15^](#_ENREF_15) | I | Single blinded randomized controlled trial | 129 people acute MI were randomized into and IV nitroglycerin group (62 patients) and a placebo group (67 patients). Outcomes: Peak CK. | Peak CK did not differ between the groups as a whole. Subgroup analysis did show non Q-wave MI patients had a significantly lower peak CK with nitroglycerin vs placebo (523 IU/l vs 1049 IU/l p=.002) | Early study unable to calculate infarct size directly, using CK as a surrogate. Single-blinded study. |
| Jugdutt 1988[^16^](#_ENREF_16) | I | Single blinded randomized controlled trial | 310 patients with acute MI were randomized to placebo versus nitroglycerin infusion for 39 hours. Outcomes: CK infarct size, LV ejection fraction and clinical variables. | Nitrate group had smaller CK measured infarct size (41 vs 55 gEq). LV ejection fraction was 22% greater (p=0.05). in-hospital mortality in nitrate group was 14 vs 26% (p=0.01) but this was only in the anterior MI subgroups. | Single-blinded study. Early study unable to calculate infarct size directly, using CK measurements as surrogate. |
| Gobel 1995[^46^](#_ENREF_46) | I | Double blinded randomized controlled trial | 129 patients with unstable angina were randomized to glyceryl trinitrate or diltiazem. Outcomes: Myocardial Infarction, refractory angina | Patients with refractory angina were smaller in diltiazem group when compared to nitrate (10 vs 18% p=.02). Infarction was 15% in diltiazem group and 38% in nitrate group, suggesting diltiazem as a better agent. | No data timing of administration. |
| Ohlin 1998[^17^](#_ENREF_17) | I | Single blinded randomized controlled trial | 43 patients receiving thrombolytic therapy for acute MI were randomized to receiving IV nitroglycerine at the same time as lytics or 3 hours after lytics.  Outcomes: Plasma levels of malondialdehyde (MDA) which correlate with successful reperfusion were measured. | Patients who received nitroglycerine concomitantly with lytics had no change in their MDA levels at 90 minutes. The group receiving nitrates 3 hours later had a significant increase in their plasma MDA levels (p=.036). This suggests nitroglycerine may have a detrimental affect on reperfusion when given with lytics. | Clinicians and patients could not have been blinded to study groups even though those measuring the MDA levels were blinded. |
| Nicolini  1994[^18^](#_ENREF_18) | II | Non-randomized Controlled Trial | 46 patients with ACS in a coronary care unit and all patients received tPA. Decision to give nitroglycerin infusion left to treating physician. Those not receiving nitrate treatment (n=11) were in group A, those receiving nitrate treatment (n=35) in group B. Those not receiving nitrates were given saline. Outcome: Plasma levels of tPA | Plasma levels of tPA were found to be significantly higher in the non-nitroglycerin group (p=.005). | No true randomization. No blinding of therapy to caregivers, patients or investigators. |

**Evidentiary Table: Morphine Sulfate**

| **Study** | **LOE** | **Study Design** | **Methods and Outcomes** | **Results** | **Limitations** |
| --- | --- | --- | --- | --- | --- |
| O’Connor 2010 for ILCOR[^5^](#_ENREF_5) | I | Comprehensive Review | Structured database review with the goal of creating guidelines for ACS care. 25 topics were selected for review including the use of opiates and other sedative/analgesics. | Minimal studies were found that looked at opiate use in ACS. A single study was noted that seemed to show that both mortality and rates of MI were more prevalent in patients with NSTEMI who received morphine. | Minimal studies noted. |
| Meine 2005[^19^](#_ENREF_19) | I | Prospective, Observational Study (cohort) | Nonrandomized, review of patients presenting at 443 hospitals in the US with ACS. 57,039 patients were evaluated and outcomes were compared between patients receiving morphine versus not, and those receiving morphine versus nitroglycerine. Oucomes: Mortality rate. | Patients receiving morphine had a higher risk of death both compared to no morphine (OR 1.48) and to those receiving nitroglycerine (OR 1.50). Morphine was associated with increased in-hospital mortality (OR 1.41). Propensity score matching was utilized. | No randomization |

**Evidentiary Table: Pre-hospital 12-lead EKG**

| **Study** | **LOE** | **Study Design** | **Methods and Outcomes** | **Results** | **Limitations** |
| --- | --- | --- | --- | --- | --- |
| O’Connor 2010 for ILCOR[^5^](#_ENREF_5) | I | Comprehensive Review | Structured database review with the goal of creating guidelines for ACS care. 25 topics were selected for review including the use of Pre-hospital and emergency department EKG in the diagnosis of STEMI. | 5 studies showing the utility of prehospital EKG and transmission. 16 studies suggesting non-physicians (paramedics and nurses) are able to accurately diagnose STEMI. Multiple studies suggesting benefit to augmenting prehospital EKG diagnosis with computer assistance. | No recommendation on prehospital right-sided EKG |
| Ionnidis 2001[^20^](#_ENREF_20) | II | Meta-analysis | Systematic review and metanalysis of all English literature between 1966 and 1998 on out-of-hospital EKG. Sensitivity, specificity and odds ratios were calculated. Outcomes: time savings, early ventricular function, early mortality, long-term survival. | Sensitivity for acute MI were 68%, specificity was 97% with diagnostic OR of 104. Conclusions are that pre-hospital 12-lead-EKG has “excellent diagnostic performance” for detecting acute MI | Prospective and retrospective non-randomized trials included in analysis. |
| Kudenchuk 1998[^21^](#_ENREF_21) | I | Prospective, observational Study (cohort) | Prehospital EKGs were obtained in 3,027 consecutive patients with symptoms of acute MI as part of the MITI project on prehospital versus hospital thrombolysis. The patients were then followed in hospital and the final hospital discharge diagnosis was used to diagnose acute MI. Outcomes: Utility of prehospital EKG in diagnosing acute MI. | ST segment and T/Q wave changes were much more prevalent on prehospital and in-hospital EKGs in those with acute MI as compared to those without. Conclusion was that prehospital EKG can identify acute MI. | Original study was looking at benefit of prehospital fibrinolysis. The EKG analysis was a subsequent study. |
| Feldman 2005[^22^](#_ENREF_22) | I | Prospective, observational Study (cohort) | Paramedics in a large urban system all underwent a 6-hour EKG training and patients with clinical suspicion of acute MI were enrolled over 8 months. EKGs were interpreted by paramedics, EM physician, cardiologist and a third, blinded physician who assigned a final diagnosis based on WHO criteria for acute MI. Outcomes: Accuracy of paramedics EKG interpretation. | 166 patients were enrolled. Paramedics correctly identified 20 of 25 patients with STEMI and incorrectly identified 4 patients as STEMI. Paramedic interpretation was comparable to blinded EM physician (94 vs 93% accuracy) and cardiologist (95%). | Single system. 6-hour EKG training may not be practical for some systems. |
| Le May 2006[^23^](#_ENREF_23) | I | Prospective, observational study (cohort) | A STEMI tool, based on 12-lead EKG, was developed and employed by an urban EMS system in Canada. Paramedics had 8-hours EKG training as part of their certificate. Paramedics then used the tool to diagnose STEMI in the field. 2 EM physicians and 1 Cardiologist independently reviewed the blinded EKGs using the same diagnostic criteria. Outcome: Accuracy of paramedic EKG interpretation of STEMI. | 967 patients were evaluated and EKGs were available in 97% of them. 411 patients had the STEMI tool. Paramedics missed STEMI in 3 patients and incorrectly assigned STEMI in 13 patients. Sensitivity for diagnosis was 95% and specificity was 96%. Conclusion: Paramedics can accurately diagnose STEMI in the field based on EKG findings. | Poor compliance with decision tool led to only 43% of patients receiving the full study protocol. |
| Van’t Hof 2006[^24^](#_ENREF_24) | I | Prospective, observational study (cohort) | Originally part of the On-TIME trial, 209 patients were included after prehospital diagnosis of acute MI compared to 258 patients who were diagnosed at a non-PCI center. Outcomes: accuracy of diagnosis, time to treatment, LV function, clinical outcome. | Patients triaged by ambulance had a correct acute MI diagnosis 95% of the time compared to 99% when coming from non-PCI hospital. Ambulance interpretation was independently associated with LVEF >40%. | No randomization. Decision to send to a non-PCI center made based on whether or not ambulance had 12-lead capabilities. |
| Millar-Craig 1997[^26^](#_ENREF_26) | II | Prospective, controlled study | Phase I: After extensive training in EKG interpretation and acute MI diagnosis, paramedics transported patients to the emergency department for evaluation, treatment and transfer to the CCU. Phase II: Paramedics that had at least 80% accuracy in EKG interpretation were allowed to transport directly to the CCU for decision on thrombolytic therapy. Outcomes: EKG diagnosis accuracy, Door-to-needle time. | Accuracy of STEMI diagnosis by paramedics was 87.5% in phase I and 92% in phase II. Call to thrombolysis time was reduced from 154 minutes to 93 minutes and door-to-needle time was reduced from 97 minutes to 37 minutes. | Non-randomized study. Significant training involved for paramedic crews may not translate easily to many EMS systems. |
| Verbeek 2012[^27^](#_ENREF_27) | II | Retrospective Observational Study | 325 charts with diagnosis of STEMI were reviewed in an urban EMS system that requires up to 3 EKGs in their chest pain protocols. Sensitivities for STEMI were calculated based on initial and subsequent EKGs. | 84.6% of STEMI were identified with the first EKG, 93.8% with first or second and 100% with first, second and third EKG. This suggests repeat EKGs are beneficial in diagnosing STEMI. | Retrospective study. Single EMS system. |
| Bradley 2006[^28^](#_ENREF_28) | III | Cross-Sectional Study | Survey developed for 365 hospitals to identify which, of 28 different strategies, was most associated with decreased doo-to-balloon times in STEMI. Multivariate regression analysis performed. | Six strategies were associated with reduced door-to-balloon time, including a 15.4 minute reduction when paramedic field report is used to activate the catheterization laboratory. | Respondents were singular individuals at the hospital and the reported policies were not verified. |
| Kudenchuk 1991[^29^](#_ENREF_29) | I | Prospective, observational study (cohort) | Prehospital EKG was obtained in 1,189 participants during an ongoing study on thrombolytic therapy. Outcomes: Accuracy of computer interpretation of EKG for STEMI. | Positive predictive value of acute MI with computer-interpretation vs physician was 94 vs 86% respectively. NPV was 81 vs 85%. Conclusion suggests prehospital screen for acute MI with computer interpretation is feasible and highly specific. | Older study, no randomization, older technology. |
| Dhruva 2007[^30^](#_ENREF_30) | II | Prospective, observational study using historical controls | The STAT-MI network was created to integrate wireless technology to transmit prehospital EKGs. Prospectively collected data from the STAT-MI network was analyzed to determine if transmission of prehospital EKGs to PCI-capable hospitals leads to a decrease in door-to-balloon times. | 80 EKGs were transmitted by the network during the study period (7 months). Door to intervention decreased to 80.1 minutes from 145.6 minutes using the historical control (prior to implementing STAT-MI). Conclusion is that prehospital transmission can decrease door-to-balloon time. | Historical control group. Non-randomized study. |

**Evidentiary Table: Regionalization of STEMI Care**

| **Study** | **LOE** | **Study Design** | **Methods and Outcomes** | **Results** | **Limitations** |
| --- | --- | --- | --- | --- | --- |
| O’Gara 2013 for the AHA[^40^](#_ENREF_40) | I | Comprehensive Review/Clinical Guideline | Comprehensive review on the treatment of ST-elevation MI, including the role of regionalized systems of STEMI care with the goal of creating practice guidelines for patients with acute MI. | Regionalized systems of care is given a class I recommendation by the AHA including recommendations for immediate transport to PCI center, if available within 120 minutes or fibrinolysis if no available PCI center. | These are clinical guidelines and not original research, though all recommendations are based on clinical studies. |
| Keeley  2003[^31^](#_ENREF_31) | I | Quantitative Review/  Metanalysis | Search of published works were performed and identified 23 randomized controlled trials of patients randomized to primary PCI vs fibrinolysis. Outcomes: Death, reinfarction, stroke. | Primary PCI more effective than fibrinolysis at reducing death (7 vs 9%), reinfarction (5 vs 7%) and stroke (1 vs 2%). Conclusions: Primary PCI is a more effective treatment than fibrinolysis for treatment of STEMI. | Not all principal investigators could be contacted and there were many variations in study protocols and patient populations. |
| McNamara 2006[^32^](#_ENREF_32) | I | Prospective, observational study (cohort) | Data from the National Registry of Myocardial Infarction was used to study a cohort of 29,222 STEMI patients who received PCI within 6 hours of presentation. Outcomes: in-hospital mortality based on door-to-balloon time. | Longer door-to-balloon time was associated with increased in-hospital mortality with the lowest mortality being within 90 minutes (3%) and highest mortality being greater than 150 minutes (7.4%). Door-to-balloon time of >90 minutes associated with increased mortality (OR 1.42; 95% CI 1.24 to 1.62). | No randomization. |
| Nallamothu 2007[^33^](#_ENREF_33) | I1 | Prospective, observational study (cohort) | Multinational study collecting data on ACS care. This is an observational study on this prospectively collected data. Primary outcome was 6-month mortality with the variable being door-to-balloon times for PCI and door-to-needle time for fibrinolysis. | 3959 total patients with 1786 (45.1 %) receiving fibrinolysis and 2173 (54.9%) receiving PCI. PCI patients had 0.18% increased mortality per 10 min delay between 90 and 150 min and fibrinolysis patients had a 0.30% increased mortality per 10 min delay between 30 and 60 min. | No randomization, missing or incomplete information and possible confounding with unmeasured covariates. |
| Langabeer 2014[^36^](#_ENREF_36) | II | Retrospective cohort | This study reviews the impact of STEMI regionalization on door-to-balloon time in an urban EMS system. 15 months of data were reviewed looking at all diagnoses of STEMI who were treated with primary PCI. The study excluded transfers. These patients were compared to those who self presented to the ED with STEMI. Outcomes: Door-to-balloon time. | 747 total patients were identified. An 11.1 minute reduction in door-to-balloon time was found with EMS transport vs self-transport. In addition, when medics activated the catheterization lab from the field, door-to-balloon times were reduced by 43 min (38%). Conclusion: EMS engagement with STEMI treatment actively decreases door-to-balloon times. | Difficult to generalize to rural populations. No randomization. |

**Evidentiary Table: Prehospital Fibrinolysis**

| **Study** | **LOE** | **Study Design** | **Methods and Outcomes** | **Results** | **Limitations** |
| --- | --- | --- | --- | --- | --- |
| FTT Collaborative Group 1994[^38^](#_ENREF_38) | I | Systematic Review/  metanalysis | 9 randomized controlled trials, randomizing more than 1000 patients to fibrinolysis versus placebo were reviewed for demographic data, clinical presentations and outcome data. Outcomes: Death, major adverse events (stroke, hemorrhage). | Absolute mortality reduction of 30 per 1000 for patients with STEMI and BBB within the first 6 hours of treatment. Excess of deaths from fibrinolysis on day 1 of treatment, but significant mortality benefit on days 2-35. | Early review, no comparison with PCI. |
| AIMS Trial Study Group[^39^](#_ENREF_39) | I | Double-blinded randomized controlled trial | 1004 patients randomized to streptokinase versus placebo. Interim mortality analysis was performed. Outcome: 30 day mortality | 61 out of 502 patients on placebo died within 30 days (12.2%) vs 32 of 502 patients who were given streptokinase (6.4%). The study was halted early due to the 47% decrease in mortality. | Full study not completed, though interim data is convincing and required stoppage of the study for ethical reasons. |
| O’Gara  2013 for the AHA[^40^](#_ENREF_40) | I | Comprehensive Review/Clinical Guidelines | Comprehensive review on the treatment of ST-elevation MI, including the role of fibrinolysis with the goal of creating practice guidelines for patients with acute MI. | Class I recommendation with Level A evidence to perform fibrinolysis on patients with STEMI when it is anticipated that PCI will not be available within 120 minutes. | These are clinical guidelines and not original research, though all recommendations are based on clinical studies. |

**Evidentiary Table: Prehospital Use of β-Blockers**

| **Study** | **LOE** | **Study Design** | **Methods and Outcomes** | | **Results** | **Limitations** |
| --- | --- | --- | --- | --- | --- | --- |
| Ibanez  2013[^41^](#_ENREF_41) | I | Single-blinded, randomized controlled trial | Patients presenting with anterior STEMI were randomized to either intravenous metoprolol (given on arrival to the ED or prehospital) or control group. All patients were then treated with standard medical therapy including PCI. Outcomes: Infarct size as measured by MRI, major cardiac events, death, malignant arrhythmias, shock, re-infarction. | Infarct size was smaller with IV metoprolo vs control (25.6 vs 32g), composite of death, cardiac events, arrhythmia and shock in metoprolol vs control was better (7.1 vs 12.3%) although not significant (p=0.21). | | Only evaluators were blinded to study groups. 19% of the patients did not undergo MRI imaging for infarct size. |
| Mateos  2015[^42^](#_ENREF_42) | I | Subgroup Analysis of Single-blinded randomized controlled trial | Subgroup analysis of patients in the METOCARD-CNIC Trial who received prehospital metoprolol versus control patients who did not receive metorprol. Outcomes: Infarct size at one week, adverse events. | 147 total patients enrolled (74 randomized to metoprolol, 73 control). Metoprolol patients had smaller infarct size (23.4 vs 34g), higher EF (48.1 vs 43.1%) and did not have increased adverse events. | | International study with physician staffed ambulances. Results may not be generalizable to U.S. EMS systems. |

AMI= Acute Myocardial Infarction, O^2^=oxygen, ACS= Acute Coronary Syndrome, STEMI=ST segment Elevation Myocardial Infarction, EKG=Electrocardiogram, LV=Left Ventricle, MRI=Magnetic Resonance Imaging, L=Liter, CK=Creatinine Kinase, MI=Myocardial Infarction, ASA=Aspirin, EMS=Emergency Medical Services, PCWP=Pulmonary Capillary Wedge Pressure, CK-MD=Creatinine Kinase MB Band, MDA=Malondialdehyde, NSTEMI=nonSTEMI, OR=Odds Ratio, EM=Emergency Medicine, WHO=World Health Organization, CCU=Cardiac Care Unit, PCI=Percutaneous Intervention, BBB=Bundle Branch Block, EF=Ejection Fraction
